# Developmental Language Disorder and Psychopathology: Disentangling Shared Genetic and Environmental Influences

**DOI:** 10.1177/00222194211019961

**Published:** 2021-06-11

**Authors:** Umar Toseeb, Olakunle Ayokunmi Oginni, Philip S. Dale

**Affiliations:** 1 University of York, UK; 2 King’s College London, UK; 3 The University of New Mexico, Albuquerque, USA

**Keywords:** developmental language disorder, heritability, mental health, psychopathology, childhood, adolescence

## Abstract

There is considerable variability in the extent to which young people with developmental language disorder (DLD) experience mental health difficulties. What drives these individual differences remains unclear. In the current article, data from the Twin Early Development Study were used to investigate the genetic and environmental influences on psychopathology in children and adolescents with DLD (*n* = 325) and those without DLD (*n* = 865). Trivariate models were fitted to investigate etiological influences on DLD and psychopathology, and bivariate heterogeneity and homogeneity models were fitted and compared to investigate quantitative differences in etiological influences on psychopathology between those with and without DLD. The genetic correlation between DLD and internalizing problems in childhood was significant, suggesting that their co-occurrence is due to common genetic influences. Similar, but nonsignificant effects were observed for externalizing problems. In addition, genetic influences on internalizing problems, but not externalizing problems, appeared to be higher in young people with DLD than those without DLD, suggesting that the presence of DLD may exacerbate genetic risk for internalizing problems. These findings indicate that genetic influences on internalizing problems may also confer susceptibility to DLD (or vice versa) and that DLD serves as an additional risk factor for those with a genetic predisposition for internalizing problems.

Mental health difficulties during childhood and adolescence are common. Half of all lifetime psychiatric illnesses have their onset before the age of 14 years (Kessler et al., 2005). Young people with developmental language disorder (DLD), a common childhood-onset disorder, are disproportionately affected by such difficulties (Conti-Ramsden & Botting, 2008). Although the overlap between DLD and psychopathology is well documented, research on the etiological influences on the relationship between the two is scarce. In the present study, behavioral genetic methods were used to help shed new light on the basis of the relationship between DLD and psychopathology during childhood and adolescence.

## Developmental Language Disorder

DLD affects approximately 5% to 7% of children who start primary school each year (Norbury et al., 2016). It is a neurodevelopmental condition that is characterized by problems with learning and using oral language. The term *developmental language disorder* is relatively new and is used to refer to young people with disordered language in the absence of certain neurodevelopmental conditions that often entail language difficulties, such as autism spectrum disorders (Bishop et al., 2017). The category of DLD is related to but not the same as that of specific language impairment (SLI). Young people with SLI are required by definition to have nonverbal cognitive ability within the normal range (Tomblin et al., 1997) whereas those with DLD may or may not have the low nonverbal cognitive ability (Bishop et al., 2017). Consequently, all young people with SLI can be referred to as having DLD but not all of those with DLD can be referred to as having SLI. Critics of the term SLI argue that it is too narrow and that nonverbal IQ does not distinguish language profiles in children with language impairments. In doing this, the narrow SLI label may exclude children with low IQ and language impairment from receiving effective support (Bishop, 2017). Evidence from twin research suggests that the SLI is not genetically distinct from non-specific language impairment (Bishop, 1994). Therefore, in this study, the definition of DLD was adopted and will be used in this article to refer to previous studies of young people with DLD or SLI.

DLD manifests in different forms, meaning that those affected have varied strengths and weaknesses within language. Young people with DLD also often experience difficulties in multiple other areas of functioning. For example, compared with their typically developing peers, young people with DLD have poorer quality friendships (Durkin & Conti-Ramsden, 2007), are more socially withdrawn (Hart et al., 2004), and are more likely to be bullied (van den Bedem et al., 2018). Such difficulties with peers are, however, not inevitable. Recent work by Toseeb et al. (2020), in a large community-based sample, found no differences in friendship quality between children with DLD and their typically developing peers. Indeed, a recent systematic review found substantial individual differences in peer interactions among children with DLD, with some displaying considerable strengths (Lloyd-Esenkaya et al., 2020).

At a group level, such difficulties continue beyond school age; young adults with DLD have higher mean levels of shyness and lower levels of self-efficacy and self-esteem compared with their typically developing peers (Botting et al., 2016; Durkin et al., 2017). Furthermore, those with DLD tend to have poorer employment and educational outcomes compared with their typically developing peers (Johnson et al., 2010), although the situation does appear to have improved in recent years, possibly due to increased access to vocational training (Conti-Ramsden et al., 2018).

## DLD and Psychopathology

Child and adolescent psychopathology can take many forms, and co-occurrence of symptoms across diagnostic categories is common. Although symptoms of internalizing (e.g., anxiety and depression) and externalizing problems (e.g., conduct problems and hyperactivity) reflect an overarching psychopathology factor (Patalay et al., 2018), these symptom domains were negatively correlated after adjusting for a general psychopathology factor (Caspi et al., 2014). This suggests that internalizing and externalizing problems can be viewed, to some extent, as different outward manifestations of a common underlying vulnerability (Sallis et al., 2019). For brevity’s sake, however, the term *psychopathology* is used in this article to refer to both internalizing and externalizing problems.

Young people with language disorders, such as DLD, have high rates of diagnosable psychopathology. Some early estimates suggest that more than 70% of young people with a language disorder have a psychiatric disorder (Cantwell & Baker, 1987). Even at a symptom level, young people with DLD have, on average, increased levels of psychopathology compared with their typically developing peers (Yew & O’Kearney, 2013). Such difficulties are not, however, inevitable and there is considerable diversity in the profiles of psychopathology in young people with DLD; some experience very few difficulties during childhood and adolescence, and others considerably more. In one study, nearly a third of young people with DLD had very few (or no) externalizing problems (Pickles et al., 2016) and in the same sample, approximately 1 in 10 had very few (or no) internalizing problems (Conti-Ramsden et al., 2019). There is also considerable co-occurrence of symptoms across diagnostic categories in those with DLD. For approximately half of young people with DLD, internalizing problems such as emotional and peer problems co-occur during development in childhood and adolescence (Conti-Ramsden et al., 2019). Externalizing problems such as conduct problems and hyperactivity also follow a common developmental trajectory for approximately three quarters of young people with DLD (Pickles et al., 2016). What predicts individual differences in psychopathology in young people and adolescents with DLD remains unclear.

## Phenotypic Associations Between DLD and Psychopathology

The focus of studies investigating these relationships among disorders has predominantly been on the identification of behavioral factors that are associated with higher or lower levels of psychopathology in young people with DLD and then attempting to make inferences about causality. For example, peer problems, bullying victimization, and maladaptive emotional regulation strategies are all associated with higher levels of internalizing problems in young people with DLD (Forrest et al., 2018, 2020; Kilpatrick et al., 2019; St Clair et al., 2019). Conversely, higher levels of prosociality, play, and emotional awareness are associated with fewer internalizing and/or externalizing problems in young people with DLD (Bakopoulou & Dockrell, 2016; Samson et al., 2020; Toseeb et al., 2017, 2020; Toseeb & St Clair, 2020). Although informative, such studies are limited in their ability to predict the direction of the observed effects. It is still not clear whether DLD leads to increased psychopathology, psychopathology leads to DLD, or there is a bidirectional effect between the two. Alternatively, it is possible that they are both caused by common genetic and/or environmental influences, or a combination of all of the above.

There are several reasons why DLD may lead to increased psychopathology. Social information processing theory suggests that children’s cognitive abilities influence their social interactions (Crick & Dodge, 1994). Indeed, positive social interactions are associated with lower levels of psychopathology in young people with DLD (Toseeb et al., 2020; Toseeb & St Clair, 2020). One possibility is that young people with DLD who are not able to successfully navigate social interactions due to language limitations become socially withdrawn, leading to higher levels of psychopathology. Furthermore, recognizing others’ emotions affects young people’s ability to understand others’ intentions, which influences their social interactions (Crick & Dodge, 1994). Young people with DLD, who often have difficulties in recognizing others’ emotions, tend to have higher levels of social anxiety leading to higher levels of psychopathology (Samson et al., 2020; van den Bedem et al., 2020). Deficits in language may entail a limited ability to appropriately recognize and label emotions. Therefore, DLD may lead to increased psychopathology via impaired social functioning.

It may also be that psychopathology, or at least earlier social and emotional difficulties, leads to or exacerbates DLD. Usage-based approaches to language acquisition suggest the importance of social context (Tomasello, 2003). Early social interactions provide opportunities to learn and practice language (Hoff, 2006). Children with higher levels of psychopathology are likely to have poorer social interactions, which may lead to impairments in language development. For example, social withdrawal at the age of 1 year is associated with subsequent delays in reaching language development milestones (Guedeney et al., 2016). Indeed, those with poorer quality relationships with their primary caregivers earlier in life tend to have language difficulties in childhood (St Clair et al., 2019). This may create fewer opportunities for language acquisition by creating a poor early language and communication environment (Gibson et al., 2020). Therefore, DLD and psychopathology may co-occur because the symptoms of psychopathology create environments and social situations that are not conducive to language acquisition and development.

Another possibility is that DLD and psychopathology may be influenced by common genetic factors. Accumulating evidence suggests that genes have generalist effects and influence multiple areas of functioning during childhood (biological pleiotropy) while environmental influences serve to distinguish between internalizing and externalizing problems (Marceau & Neiderhiser, 2020). Alternatively, mediated pleiotropy may be at play whereby genetic influences on DLD may be transmitted to psychopathology through a phenotypic association between DLD and psychopathology or vice versa (Wedow et al., 2018). That is, genetic factors influence the onset of DLD which then leads to increases in psychopathology or, alternatively, genetic factors increase the risk of psychopathology which then leads to DLD.

Therefore, a reasonable starting point for understanding the aetiological relationship between DLD and psychopathology is to investigate the extent to which the genetic and nongenetic (environmental) influences on DLD and psychopathology are shared or correlated.

## Behavioral Genetics

Behavioral genetic methods can be used to investigate the genetic and environmental influences on any given behavior (Rijsdijk & Sham, 2002). Almost all psychological traits are at least partly heritable (Turkheimer, 2000). Behavioral genetic methods parse variance in a trait, or the covariance between two or more traits, into genetic and nongenetic (environmental) influences. One such method is the classical twin design, which compares similarities and differences between identical (monozygotic [MZ]) and nonidentical (dizygotic [DZ]) twins (Rijsdijk & Sham, 2002). To the best of the authors’ knowledge, only two studies have investigated the genetic and environmental influences on the phenotypic correlation between language ability (or indeed language difficulties) and psychopathology. The first study used a statistical approach known as polygenic scoring to estimate whether groups of genetic variants, identified directly from DNA, which are associated with language-related difficulties also predict psychopathology in a community-based sample of children (Newbury et al., 2019). These researchers found some evidence of a genetic correlation between language ability and internalizing problems (specifically peer problems) but the effect size was very small (variance explained <0.3%) as is typical of the analytical approach (Lewis & Vassos, 2020). The second study used a sibling design to estimate the extent to which language difficulties and internalizing problems are due to common genetic and/or shared environmental influences (Helland et al., 2020). They found that common familial influences explained most of the phenotypic correlation between language difficulties and psychopathology. However, the design of the study meant that familial influences could not be further parsed into additive genetic and shared environmental effects (such as the home environment). Therefore, the previous research on this topic is limited by design constraints. Considering the different etiological and symptom profiles of internalizing and externalizing problems, it is notable that no studies have comparatively investigated the etiological overlap between DLD and both symptom domains. Furthermore, as novel genetic and environmental influences on psychopathology emerge during development, in addition to those manifesting earlier in childhood (Nivard et al., 2015), it will be informative to investigate the extent to which these novel influences overlap with those on DLD. For example, etiological influences on DLD may correlate differently with influences on psychopathology that are active in childhood compared with those that emerge in adolescence. Such knowledge can help identify periods during development that interventions may be most beneficial.

An alternative explanation for the etiological association between DLD and psychopathology in young people is that genetic and/or environmental influences on psychopathology are stronger in the presence of DLD. For example, DLD may create a stressful environment, which can potentiate genetic influences on psychopathology as captured by the diathesis-stress framework (Manuck & McCaffery, 2014; Uher, 2014); however, this possibility has not been previously investigated.

Distinguishing between shared and unique genetic or environmental influences is important as it may help to inform interventions aimed at ameliorating the effects of both DLD and psychopathology. If DLD and psychopathology are influenced by shared genetic or environmental factors, a common set of interventions could be used to target both areas of functioning. There is already some evidence for this from behavioral studies whereby positive early language and communication environments are associated with *both* better subsequent language development *and* lower levels of psychopathology in young people with DLD (Toseeb et al., 2020). Understanding this will help to shed new light on why some young people with DLD have lower levels of psychopathology than others and inform future research on targeting limited resources at the most vulnerable young children.

## The Current Study

For the first time, in the present study, the genetic and environmental influences on psychopathology in young people with and without DLD were systematically investigated and compared. The study was motivated by two key research questions.

**Research Question 1 (RQ1):** To what extent is the co-occurrence of DLD and psychopathology due to shared genetic and environmental influences?

Our first hypothesis was that:

Hypothesis 1 (H1): Psychopathology and DLD are correlated at the phenotypic level because they share both common genetic and environmental influences.

It was expected that there would be some shared etiological influences between DLD and psychopathology based on previous work (Helland et al., 2020; Newbury et al., 2019). Second, we were interested in whether there are quantitative differences in the etiological influences on psychopathology between young people with or without DLD; that is:

**Research Question 2 (RQ2):** Are the relevant genetic and environmental influences on internalizing and externalizing problems larger or smaller in young people with and without DLD?

As this question has not been previously investigated, this analysis was exploratory. However, considering evidence of genetic correlations between psychopathologies including in those with language difficulties (Allegrini et al., 2020; Helland et al., 2020) and the high rate of psychopathology in children with DLD (St Clair et al., 2011), it is likely that genetic influences on psychopathology are higher among young people with DLD compared with their typically developing peers.

## Method

### Study Sample

**Figure 1.**
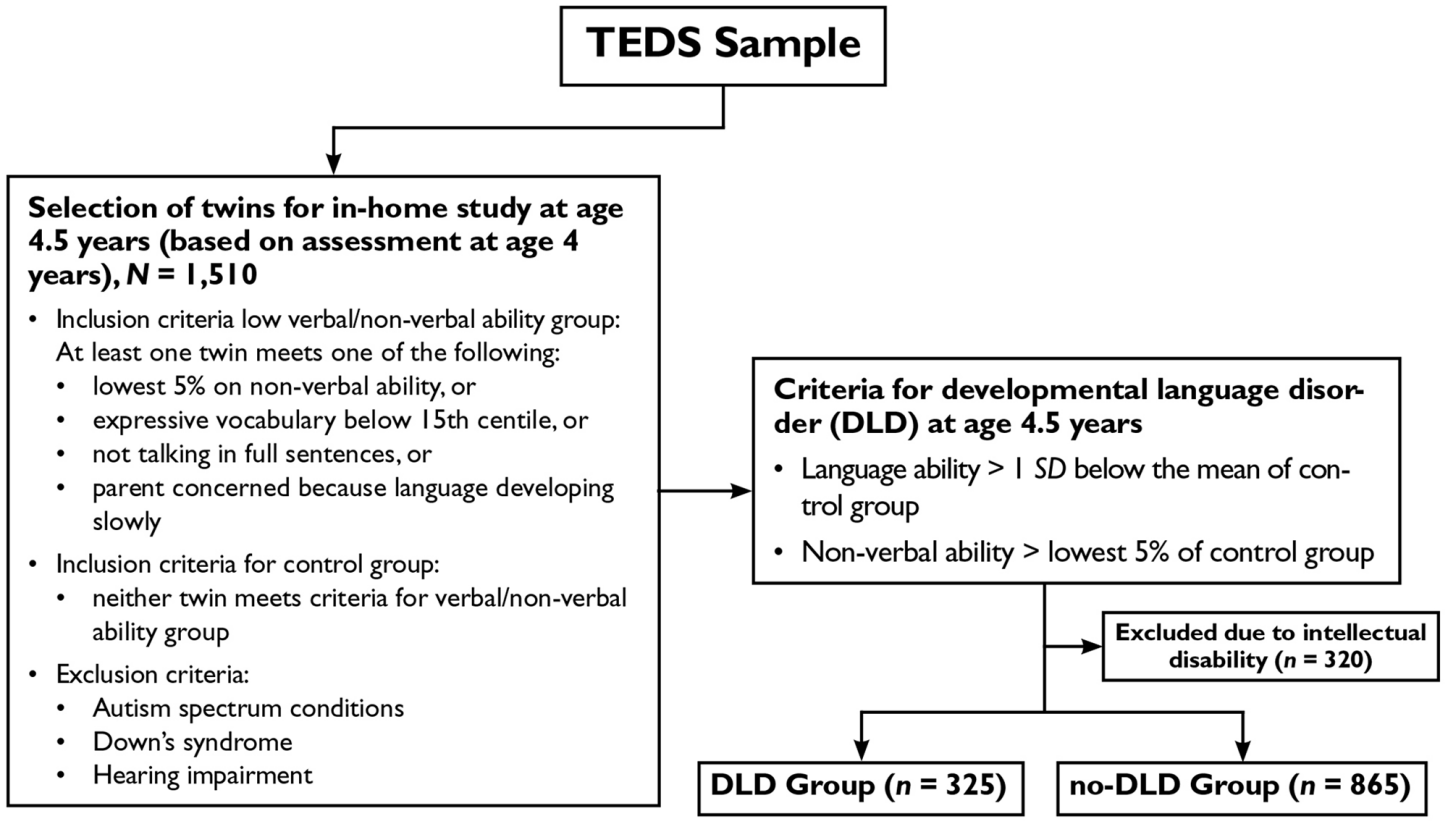
Identifying young people with developmental language disorder from the TEDS sample. TEDS = Twins Early Development Study.

Data from the Twins Early Development Study (TEDS) were used in the current analyses. TEDS is a longitudinal twin cohort study of children born in England and Wales between 1994 and 1996 (Haworth et al., 2013). Nearly 14,000 families took part in the first wave of data collection when the twins were 18 months old. Subsets of the original sample were invited to take part in subsequent waves. At the time of recruitment, the sample was representative of the U.K. population. Full details of the full TEDS sample representativeness are described elsewhere (Rimfeld et al., 2019). The analysis reported here focused on the subsample of twins who took part in an additional in-home study, which was no longer representative of the U.K. population. Families were invited to take part in the in-home study when the twins were 4.5 years old based on parent-reported verbal and nonverbal ability at age 4 years. For ease of comprehension, both of these time points are collectively referred to as childhood. The criteria for inclusion in the in-home study are shown in Figure 1.

Seven hundred and fifty-five families took part in the in-home assessment (mean age = 4.51 years, *SD* = 0.20 years). The sample consisted of 281 MZ twin pairs and 474 DZ twin pairs. For 50% of the families, at least one twin had low language or nonverbal ability (as shown in Figure 1). For the remaining 50%, neither twin had low language nor non-verbal ability (referred to as the control group).

#### Developmental language disorder status

DLD status was determined in childhood using measures administered during the in-home assessments. Young people were categorized as having DLD if they scored > 1*SD* below the mean of the control group on the language composite (described below) in the absence of intellectual disability (lowest 5% nonverbal ability composite, described below). This yielded a sample of 325 young people with DLD and 865 without DLD.

### Measures

#### Language composite

A comprehensive battery of language assessments was administered. The language composite in this sample was created in the same way as previous research (Bishop & Hayiou-Thomas, 2008), by averaging *z-*scores (computed relative to the control group) from seven measures of language skills. The measures have previously been shown to load on to a single factor (Hayiou-Thomas et al., 2006). They were as follows:

1. Bus Story Test (Renfrew, 1997): The information score was used to measure language comprehension and expression. The researcher read a story from a book with pictures and the task was for the child to retell the story using the pictures. The information score was used whereby the child receives points for content rather than grammatical complexity (e.g., 1 point for mentioning the policeman, 1 for mentioning the whistle, and another for mentioning what the policeman said).
2. Action Picture Test (Renfrew, 1997): The grammar index was used to measure the production of inflectional morphology. The child was presented with 10 picture cards and asked to describe each one (e.g., the card shows a girl cuddling a teddy bear, and the child receives a point for using “-ing” on “cuddling”).
3. British Ability Scales (Elliot et al., 1996): A score was derived using only the items that require syntactic comprehension (as opposed to purely lexical). The child was presented with a set of toys and was asked to arrange them according to the researcher’s instructions (e.g., “Put the house on each side of the car.” The child received a point for each correct response).
4. Phonological Awareness Test (Bird et al., 1995) was used to measure receptive phonology. The researcher presented the child with puppets and said that the puppets like things that sound like their names. Four pictures of items with names were presented to the child, who was asked to select one (e.g., “Which of these things would Lynn like: Chair? Bin?” and so forth. The child was awarded 1 point for each correct answer.).
5. 5-7. Three subtests of the McCarthy Scales of Children’s Abilities (MCSA: McCarthy, 1972) were used: Word Knowledge (point to the picture corresponding to the word the researcher says, for example, towel), Verbal Fluency (name as many items belonging to a given category within 20 s, for example, animals), and Opposite Analogies.

#### Nonverbal ability composite

A nonverbal ability composite was generated by averaging *z-*scores from four nonverbal measures from the MCSA (McCarthy, 1972): block building, puzzle solving, tapping sequence, and draw-a-design. These four measures have previously been shown to load on to a nonverbal factor in the same sample (Viding et al., 2003).

#### Psychopathology

The parent-report Strengths and Difficulties Questionnaire (Goodman, 1997) was completed by parents about their children during childhood (mean age = 4.02 years, *SD* = 0.09 years) and adolescence (mean age = 11.34 years, *SD* = 0.67 years). There were higher levels of missing data in adolescence than in childhood (as described in the descriptive statistics section). In line with scoring guidelines (sdqinfo.org), an internalizing score (sum of emotional and peer problems subscale) and an externalizing score (sum of conduct problems and hyperactivity subscale) were created by summing the responses to the relevant items. The internal consistency of both subscales in childhood and adolescence in the study sample was acceptable (Cronbach’s αs = .60–.79). Although it is acknowledged the peer problems may occur across both internalizing and externalizing problems, the decision was taken to include peer problems in the internalizing problems score for two reasons: (a) this is in line with the scoring guidelines and (b) to allow for consistency with previous work in samples of young people with DLD (Conti-Ramsden et al., 2019; Newbury et al., 2019; Pickles et al., 2016; Toseeb et al., 2020). The limitations of this approach are highlighted in the discussion section.

### Procedure

Parents provided written informed consent for all young people who took part in the study. For the in-home assessment, a pair of researchers visited each family and each researcher tested one of the twins. That is, twins were tested simultaneously and independently by the two researchers. If hearing difficulties were suspected by the researcher, a hearing test was administered and, if confirmed, the family was labeled as a medical exclusion, and subsequently removed from the analysis reported here. Parents completed questionnaires while the young people took part in the assessments.

### Statistical Analysis

#### Data preparation

Data preparation was done in Stata/MP 16.0 (StataCorp, 2019) and the genetic analyses were run in OpenMx (Neale et al., 2016), which uses maximum likelihood (ML) estimation. The effects of confounders (age and sex) on the categorical (DLD) and continuous (psychopathology) variables was controlled by including the main effects in the threshold model of the former, and by regressing the latter on the confounders and storing the residuals. This is a standard process used to control for confounding on the average effects while the focus of analyses is on individual differences (McGue & Bouchard, 1984). The residuals were then log-transformed to normalize the distribution of the data to ensure compatibility with parametric ML estimation. Goodness of fit was determined by comparing chi-square values and degrees of freedom between models. Increases in log-likelihood indicated decreases in model fit and level of statistical significance was set at *p* < .05. Statistical significance of the estimated parameters was determined by inspecting the 95% confidence intervals.

#### The twin method

The classical twin design partitions trait variances and covariances into additive genetic (A) and shared (C) and nonshared (E) environmental influences by comparing within-pair MZ and DZ correlations in twins raised together. Standardized A, commonly referred to as heritability, is the extent to which differences between individuals are due to genetic differences. The shared environment relates to the aspects of the environment shared by twins that make them similar, such as parenting and home environment; whereas the nonshared environment refers to environmental factors not shared by twins that make them different from one another, such as peer relationships, random biological noise during development, and measurement error (Rijsdijk & Sham, 2002). MZ twins share close to 100% of their DNA while DZ twins share about 50% of theirs and, if raised together, share 100% of their common environments. Thus, any differences between MZ twin pairs reflect experiences that are unique to each individual (i.e., E influences). Higher similarities within MZ compared with DZ twin pairs suggest A influences while similar levels of similarities suggest C influences.

#### Descriptive statistics

Phenotypic correlations between DLD and psychopathology were estimated using ML estimation. Consistent with assumptions of genetic models, means and within-person correlations were constrained to be equal across birth order and zygosity. This enabled the use of a reduced set of statistics to derive multiple estimates in a genetic model.

#### Etiological influences on DLD and psychopathology (RQ1)

To investigate the shared etiological influences on the correlations between DLD and psychopathology in childhood and adolescence, two separate trivariate (DLD, psychopathology in childhood, and psychopathology in adolescence) Additive genetic (A), shared (C), and non-shared (E) environmental influences (ACE) models were specified for internalizing and externalizing problems respectively. Cholesky decomposition was used to estimate ACE parameters and interpreted using a standardized solution (Loehlin, 1996). The ACE model was specified as the cross-twin–within-trait correlations in DZ twin pairs were more than half the corresponding correlations in MZ twin pairs. This result indicates the importance of C influences rather than dominant genetic (D) influences, which would be indicated if the DZ cross-twin within-trait correlations had been less than half the MZ estimates, in which case an ADE model would be indicated. In the first model, shared etiological influences between DLD, internalizing problems in childhood, and internalizing problems in adolescence were investigated (Model 1). In the second model, shared etiological influences between DLD, externalizing problems in childhood, and externalizing problems in adolescence were investigated (Model 2). As is usually done for categorical variables, a liability threshold model was specified for DLD, with the threshold constrained to 1.48, based on a population prevalence of 5% to 7% for DLD. This model assumes a normally distributed latent liability underlying an observed categorical outcome (Neale, 2005).

#### Quantitative differences in etiological influences (RQ2)

Two bivariate heterogeneity models were run to determine if there were quantitative differences in the genetic, shared environmental, and nonshared environmental influences (i.e., differences in the magnitude of these influences in young people with or without DLD). In the first model (Model 3), the variables of interest were internalizing problems in childhood and adolescence and their covariance (continuity). In the second model (Model 4), the focus was on externalizing problems in childhood and adolescence and their covariance (continuity). In the first step of analyses, the ACE influences were allowed to differ in young people with DLD and in those without DLD (heterogeneity models) and in a subsequent step, these influences were constrained to be equal in both groups (homogeneity models). Then chi-square tests were used to determine whether model fit was significantly reduced in the homogeneity models. If the model fit of the homogeneity model is not significantly worse than the heterogeneity model, it suggests that heritability estimates are comparable in both groups.

#### Preregistration of analysis

The main genetic analyses were preregistered on the open science framework (https://osf.io/a6wf2). The analyses reported here deviated from the analysis plan as follows: After accessing the data, a decision was made to make use of the longitudinal nature of the data to investigate etiological influences on continuity of psychopathology and whether the magnitude of this differed by the presence of DLD (i.e., trivariate models were fitted instead of the planned bivariate models).

## Results

### Descriptive Statistics

The means and standard deviations (split by zygosity) are shown in Table 1. The within-person phenotypic correlations between the variables of interest are shown in Table 2. As expected, DLD was associated with higher levels of internalizing and externalizing problems both in childhood and adolescence. Cross-twin correlations were then calculated and are presented in Table 3. As expected, the cross-twin within trait correlations for all measures were higher in MZ twins than DZ twins suggesting additive genetic influences on these traits.

**Table 1.** Descriptive Statistics for Psychopathology Split by Zygosity.

Phenotype	Range	Overall		Monozygotic twins		Dizygotic twins	
		*N*	*M* (*SD*)	*n*	*M* (*SD*)	*n*	*M* (*SD*)
Childhood							
Internalizing Problems	0–15	1,174	3.08 (2.39)	427	2.89 (2.13)	747	3.19 (2.52)
Externalizing Problems	0–20	1,175	6.30 (3.36)	427	6.39 (3.00)	748	6.25 (3.55)
Adolescence							
Internalizing Problems	0–15	860	2.84 (2.79)	324	2.54 (2.69)	536	3.02 (2.83)
Externalizing Problems	0–18	860	4.25 (3.27)	324	4.18 (3.07)	536	4.28 (3.39)

*Note.* *N* = sample size, *M* = mean, *SD* = standard deviation.

**Table 2.** Within-Person Phenotypic Correlations.

Phenotype	1.	2.	3.
1. Developmental language disorder (Ch.)	1		
2. Internalizing problems (Ch.)	**.22 [.14, .29]**	1	
3. Internalizing problems (Ad.)	**.17 [.07, .26]**	**.35 [.28, .41]**	1
1. Developmental language disorder (Ch.)	1		
2. Externalizing problems (Ch.)	**.22 [.14, .30]**	1	
3. Externalizing problems (Ad.)	**.23 [.14, .32]**	**.45 [.38, .50]**	1

*Note*. Values represent correlation coefficients [95% confidence intervals]. Bold values indicate that the 95% confidence intervals did not cross zero. Ch. = childhood, Ad. = adolescence.

**Table 3.** Cross-Twin Correlations Monozygotic and Dizygotic Twin Pairs.

	Twin 2					
	Monozygotic			Dizygotic		
Phenotype	1.	2.	3.	1.	2.	3.
Twin 1						
1. Developmental language disorder (Ch.)	**.69 [.53, .81]**	—	—	**.58 [.43, .70]**	—	—
2. Internalizing problems (Ch.)	**.21 [.11, .31]**	**.68 [.61, .74]**	—	.06 [−.03, .15]	**.34 [.26, .43]**	—
3. Internalizing problems (Ad.)	.11 [−.01, .22]	**.32 [.25, .40]**	**.74 [.67, .79]**	.10 [−.01, .21]	**.19 [.11, .26]**	**.40 [.30, .50]**
1. Developmental language disorder (Ch.)	**.69 [.53, .81]**	—	—	**.58 [.43, .70]**	—	—
2. Externalizing problems (Ch.)	**.18 [.08, .29]**	**.64 [.55, .71]**	—	**.10 [.01, .19]**	**.16 [.07, .25]**	—
3. Externalizing problems (Ad.)	**.20 [.09, .31]**	**.35 [.28, .43]**	**.77 [.71, .82]**	**.15 [.04, .25]**	**.20 [.12, .28]**	**.38 [.28, .47]**

*Note.* Values represent correlation coefficients [95% confidence intervals]. Bold values indicate that the 95% confidence intervals did not cross zero. Monozygotic and dizygotic indicate separate cross-twin correlations for monozygotic and dizygotic twin pairs (Twins 1 and 2), respectively. Ch. = childhood, Ad. = adolescence.

### Etiological Influences on DLD and Psychopathology

The standardized ACE variance component estimates for DLD and psychopathology are shown in Table 4. Standardized estimates of A, C, and E influences (a^2^, c^2^, and e^2^, respectively) on each of the variables are shown in the diagonals, and influences on their covariances (i.e., shared influences) are indicated by off-diagonals. As expected, all traits were heritable to varying degrees. Between 21% and 22% of variance in DLD in childhood was due to genetic influences, 47% to 48% was due to shared environmental influences, and the remainder 31% was due to nonshared environmental influences.

**Table 4.** Standardized Variance Component Estimates for ACE Influences and 95% Confidence Intervals.

	a^2^			c^2^			e^2^		
Phenotype	1.	2.	3.	1.	2.	3.	1.	2.	3.
Model 1									
1. Developmental language disorder (Ch.)	**.22** [.02, .58]			**.47** [.17, .66]			**.31** [.19, .41]		
2. Internalizing problems (Ch.)	**1.41** [.56, 2.00]	**.66** [.47, .73]		−.43 [−1.22, .24]	.02 [.00, .17]		.03 [−.33, .35]	**.32** [.26, .39]	
3. Internalizing problems (Ad.)	.16 [-1.73, 1.56]	**.83** [.52, 1.04]	**.67** [.46, .79]	.51 [−.71,1.93]	.10 [−.04, .36]	.07 [.00, .25]	.34 [−.14, 1.02]	.06 [−.06, .20]	**.26** [.18, .29]
Model 2									
1. Developmental language disorder (Ch.)	.21 [.00, .59]	—	—	**.48** [.16, .69]	—	—	**.31** [.19, .46]	—	—
2. Externalizing problems (Ch.)	.63 [−.25, 1.50]	**.56** [.44, .65]	—	.19 [−.42, .80]	.00 [.00, .07]	—	.18 [−.23, .62]	**.44** [.35, .54]	—
3. Externalizing problems (Ad.)	.49 [−.46, 1.50]	**.79** [.58, .92]	**.75** [.63, .81]	.39 [−.44, 1.12]	.02 [−.05, .16]	.02 [.00, .12]	.13 [−.22, .49]	**.19** [.08, .33]	**.23** [.18, .29]

*Note.* Values represent correlation coefficients [95% confidence intervals]. Bold values indicate that the 95% confidence intervals did not cross zero. a^2^, c^2^, and e^2^ = standardized proportions of additive genetic, and shared and nonshared environmental influences, respectively, on DLD, internalizing and externalizing problems in childhood and adolescence (diagonals), and their covariances (off-diagonals). Negative values in the off-diagonals indicate null (0) effects, while values greater than 1 indicate 1 (100% influence of the corresponding variance component). Ch. = childhood, Ad. = adolescence.

For internalizing problems (Table 4: Model 1), the contributions of genetic and environmental influences were similar in both childhood and adolescence. Between 66% and 67% of variance in internalizing problems in childhood and adolescence was due to genetic influences and 26% to 32% was due to the nonshared environmental influences. The confidence intervals for the estimates of shared environmental influences crossed zero, suggesting that the effects of the shared environment on internalizing problems in childhood and adolescence were not statistically significant. The phenotypic relationship between DLD and internalizing problems in childhood was entirely due to common genetic influences (Table 4: Model 1: 1.41). There was, however, no significant overlap in etiological influences between DLD and internalizing problems in adolescence (Table 4: Model 1: 0.16). Despite this, the continuity between internalizing problems was also driven by genetic influences (Table 4: Model 1: 0.83). This suggests the possibility that shared genetic influences on DLD and internalizing problems in childhood carry on into adolescence while new genetic and nonshared environmental influences on internalizing problems further emerge in adolescence.

For externalizing problems (Table 4: Model 2), the magnitude of genetic and environmental influences was less similar between childhood and adolescence. In childhood, 56% of the variance in externalizing problems was due to genetic influences (compared with 75% in adolescence) and 44% due to nonshared environmental influences (compared with 23% in adolescence). Similar to the findings for internalizing problems, there were no effects of shared environmental influences on externalizing problems either in childhood or adolescence. In contrast to the finding for internalizing problems, all of the confidence intervals for etiological influences on the covariances between DLD and externalizing problems at both time points crossed zero. Although this may suggest that there was no significant overlap in etiology between DLD and externalizing disorders, the wide confidence intervals suggest low power to detect these effects. The continuity between externalizing problems at both time points was mostly driven by genetic influences (79%), while nonshared environmental influences were relatively small (19%).

### Quantitative Differences in Etiological Influences on Internalizing and Externalizing Problems

The genetic and environmental influences on internalizing and externalizing problems, split by DLD status, are shown in Table 5 (Models 3 and 4). For internalizing problems, the ACE heterogeneity model (Table 5: Model 3) had a significantly better fit than the ACE homogeneity model (i.e., when corresponding paths were constrained to be equal across groups), χ^2^(9) = 55.81, *p* < .001). Although this appears to be due to shared environmental and genetic influences on internalizing problems in childhood being, respectively, larger in young people with and without DLD, it is unusual for shared environmental influences to be so large. One possibility is that analysis by group (young people with or without DLD) essentially reduces the sample size per group, which may limit the power to separate familial influences into A and C components as has been demonstrated in previous research (Neale et al., 1994).

**Table 5.** Differences in Aetiological Influences on Internalizing and Externalizing Problems by DLD Status (Bivariate Heterogeneity Models).

	Without DLD						With DLD					
	a^2^		c^2^		e^2^		a^2^		c^2^		e^2^	
Phenotype	1.	2.	1.	2.	1.	2.	1.	2.	1.	2.	1.	2.
**Model 3**												
1. Internalizing problems (Ch.)	**.63 [.41, .72]**	—	.00 [.00, .17]	—	**.37[.28, .31]**	—	.00[.00, .38]	—	**.64[.32, .75]**	—	**.36[.22, .50]**	—
2. Internalizing problems (Ad.)	**.95 [.44, 1.29]**	**.69** [.46, .78]	.00[−.18, .38]	.00[.00, .19]	.05[−.18, .31]	**.31[.22, .42]**	−.03[−1.39 1.02]	**.63[.22, .83]**	**1.02**[.09, 2.23]	.16[.00, .53]	.02[−.36, .36]	**.20[.12, .36]**
**Model 4**												
1. Externalizing problems (Ch.)	**.57 [.40, .68]**	—	.00 [.00, .10]	—	**.43[.32, .57]**	—	.38[.00, .63]		.05[.00, .39]		**.57[.36, .86]**	
2. Externalizing problems (Ad.)	**.68 [.20, .87]**	**.71** [.41, .79]	.00 [.00, .10]	.00[.00, .24]	**.32[.15, .60]**	**.29[.21, .40]**	.66[−.29, 1.31]	.44[.00, .80]	.22[−.27, .93]	.23[.00, .64]	.12[−.25, .56]	**.33[.19, .57]**

*Note*. Values represent correlation coefficients [95% confidence intervals]. Bold values indicate that the 95% confidence intervals did not cross zero. a^2^, c^2^, and e^2^ = Standardized proportions of additive genetic, and shared and nonshared environmental influences, respectively, on DLD, internalizing and externalizing problems in childhood and adolescence (diagonals), and their covariances (off-diagonals). Negative values in the off-diagonals indicate null (0) effects, while values greater than 1 indicate 1 (100% influence of the corresponding variance component). DLD = developmental language disorder, Ch. = childhood, Ad. = adolescence.

To investigate this further, a heterogeneity submodel, testing for only genetic and non-shared environmental influences (i.e., an AE model in which shared environmental influences were dropped) was specified for internalizing problems (see Supplementary Materials). When comparing the ACE heterogeneity model to the AE heterogeneity model, the loss in fit was not significant, χ^2^(6) = 12.13, *p* = .06, suggesting that some A influences on childhood internalizing problems among young people with DLD had wrongfully apportioned as C influences. Thereafter, this heterogeneity AE submodel was compared with a homogeneity AE submodel with a significant loss in fit in the latter model, χ^2^(6) = 45.25, *p* < .001). This appeared to be mostly driven by larger A influences on internalizing problems in childhood and adolescence among young people with DLD and by correspondingly larger E influences in young people without DLD (online supplemental Table A1). Although the significant worsening of fit of the homogeneity model suggests quantitative differences in etiological influences between groups, the overlapping confidence intervals for all the parameters indicate the need for a larger sample size. Nonetheless, this suggests that the magnitude of genetic influences on internalizing problems was larger in young people with DLD compared with those without DLD.

For externalizing problems, an ACE heterogeneity model was similarly compared with an ACE homogeneity model. In contrast to that for internalizing problems, the model fit for the ACE heterogeneity model (Table 5: Model 4) was not significantly different from the ACE homogeneity model (i.e., when the A, C, and E influences were constrained to be equal in young people with and without DLD), χ^2^(9) = 7.57, *p* = .578. This suggests that the magnitude of genetic and environmental influences on externalizing problems was comparable in young people with and without DLD.

## Discussion

In this study of young people with and without DLD, the etiological influences on DLD and psychopathology were investigated. We were specifically interested in (a) the extent to which the co-occurrence of DLD and psychopathology is due to shared etiological influences and (b) whether the magnitude of etiological influences on psychopathology differs between young people with and without DLD. We found that there are common genetic influences on DLD and internalizing problems in childhood (and possibly adolescence). Although the corresponding effects for externalizing problems were substantial, these did not attain statistical significance. We also found some preliminary evidence to suggest that genetic influences on internalizing problems (but not externalizing problems) are larger in young people with DLD compared with those without DLD. These findings are discussed with reference to previous literature and relevant caveats in the subsequent sections.

### Etiological Influences on DLD and Psychopathology

In line with our predictions, our results suggest that, in childhood, DLD and internalizing problems co-occur due to shared genetic effects. In other words, the genetic influences on internalizing problems may also confer susceptibility to DLD or vice versa. For externalizing problems, while not statistically significant, the point estimates suggest that the DLD and externalizing problems may also co-occur, at least in part due to shared genetic influences. There are two possible reasons why there may be a genetic correlation between DLD and psychopathology: biological pleiotropy and mediated pleiotropy (Wedow et al., 2018). The former indicates that common genetic variants influence both DLD and psychopathology directly, while the latter indicates that genetic influences on DLD may be transmitted to psychopathology through a phenotypic association between DLD and psychopathology or vice versa.

In demonstrating the presence of these common genetic effects in childhood, the findings from the present study reinforce the need for continued exploration of the mechanisms of phenotypic correlations between DLD and psychopathology, using other behavioral genetic approaches. This research may eventually enable the use of genetic risk for one trait to predict susceptibility to the other. One such approach is genome-wide polygenic scores (indices of genetic susceptibility), which have been shown to have some utility in identifying groups of people at increased risk for psychopathology (Demontis et al., 2019; Howard et al., 2019). At present, these scores are able to identify group-level risk to varying degrees (Duncan et al., 2019) but have limited utility in predicting individualized risk (Morris et al., 2020). Therefore, future work should consider using polygenic scores of risk for psychopathology to predict susceptibility to DLD. If successful, this may aid in the early identification of those at risk of DLD and allow for targeted early interventions.

In the current study, there were no statistically significant shared etiological influences between DLD and psychopathology in adolescence. The large genetic overlap between psychopathology at both time points (~80%) suggests that genetic influences on psychopathology in childhood continue to influence psychopathology in adolescence as has been demonstrated in another study (Lewis & Plomin, 2015). Genetic correlations <1 suggest that new genetic influences emerge in adolescence. Thus, we interpret our findings as suggesting that although common genetic variants contribute to psychopathology in childhood and adolescence, only those operative in childhood are related to DLD. Nonshared environmental influences also emerge in adolescence. It is also possible that the small sample size of young people with DLD and the categorical nature of the DLD assessment may have limited the statistical power to detect an effect. Future work should consider the temporal nature of these effects in larger sample sizes with more power.

The heritability estimates for DLD and psychopathology were in the expected ranges. DLD was moderately heritable at approximately 22%. Intuitively, this may appear quite low, but previous work in this sample has shown that the heritability of DLD depends on the diagnostic criteria (Bishop & Hayiou-Thomas, 2008). Criteria that consider parental concerns (in contrast to those used in the present study) lead to higher heritability estimates (the clinical concern hypothesis). The intuitive explanation that children who are noticed by parents and referred to clinical services may have more severe language impairment is in fact not supported in the literature. Instead, those with speech production difficulties are more likely to be referred to specialist services (Zhang & Tomblin, 2000) and, therefore, represent a qualitatively different sample compared with those identified solely using psychometric language assessments. Indeed, Bishop and Hayiou-Thomas (2008) found that heritability estimates were higher in clinically referred cases compared with those identified solely using clinical language assessments. It is also relevant that speech measures generally have higher heritability than language measures (Hayiou-Thomas et al., 2006). The criteria used here for language were chosen to maximize power given the limited sample size and based on other work in this sample (Hayiou-Thomas et al., 2005). The heritability estimates for internalizing (66%–67%) and externalizing problems (56%–75%) were in line with previous work (Lewis & Plomin, 2015; Porsch et al., 2016), thus providing confidence in the analyses reported here.

### Quantitative Differences in Etiological Influences Between Groups

There were mixed findings with regard to the difference in magnitude of etiological influences between the DLD and non-DLD groups. For internalizing problems, there was some indication that the genetic influences are larger among young people with DLD compared with those without DLD. This suggests that young people with DLD may be more susceptible to genetic influences on internalizing problems compared with those without DLD. For example, the stress of coping with DLD may exacerbate genetic influences on internalizing problems (i.e., DLD moderates genetic risk for internalizing problems). This is consistent with evidence that childhood stressful experiences increase susceptibility to genetic risk for emotional problems such as depression (Uher, 2014). This possibility, however, needs to be specifically investigated, for example using genomic methods. In contrast, the etiological influences on externalizing problems appeared independent of DLD status. It is possible that these influences are less susceptible to moderation or that DLD does not specifically moderate the etiological influences on externalizing problems.

### Strengths, Limitations, and Future Directions

A major strength of the study reported here is the diverse sample. Studies with clinical populations are prone to referral bias such that young people with the most severe needs or a specific profile of difficulties are identified to receive clinical support and thus are the most likely to be referred for participation in studies. Samples derived from community-based studies allow for more broad estimates by including young people with DLD with a wider range of strengths and difficulties. In addition to this, the young people in this sample took part in a comprehensive battery of language assessments allowing for greater specificity in those young people with DLD.

Despite these strengths, there are some limitations that should be borne in mind when interpreting the findings. The sample of young people with DLD is small for behavioral genetics research. This is despite making use of one of the largest twin cohorts in the world, with well-defined language phenotypes. The sample size constraints were evident from the wide confidence intervals for some of the estimates, suggesting that the study may have been underpowered for some of the analyses. Furthermore, it is well documented that the rate of language development is slower for twins compared with singletons (Dʼhaeseleer et al., 2016; Rice et al., 2014), and this may limit the generalizability of the study findings. In addition to this, the limited sample size meant that it was not possible to investigate sex differences. This may be an important consideration given that the manifestation of psychopathology differs between males and females. The cut-off for inclusion in the DLD group was broad (>1*SD* below the mean), which was set to be consistent with previous work with this sample and also to maximize power. This may have biased the estimates as it may mean that group-level differences may have been smaller. Finally, peer problems were included only in internalizing problems, even though they may be indicative of nonspecific symptoms that are also present in externalizing problems. This may have confounded the estimates.

Future work could overcome these drawbacks in a number of ways. It may be possible to combine data from multiple twin cohorts to create a larger sample of young people with DLD. Although it might be difficult to align language phenotypes across cohorts, the loss of specificity could be offset by the increase in power. Alternatively, it may be possible to use single nucleotide polymorphism-based methods to estimate heritability. This would negate the need for twin cohorts, and thus increase the potential sample sizes available, as well as overcome concerns regarding the generalizability of the findings beyond twins. Power could also be increased by using a continuous rather than a categorical language phenotype as used in this study. The genetic relationship between DLD and psychopathology can be further investigated using more specialized methods such as Mendelian randomization, which can help determine the presence of mediated pleiotropy (i.e., determine whether the observed genetic correlations between DLD and psychopathology result from phenotypic causal relationships). Finally, given that the findings of the current study demonstrate shared etiological influences between DLD and psychopathology, future work should consider whether genome-wide polygenic scores for psychopathology can be used to identify groups of young people at risk of DLD.

## Conclusion

Overall, the current study adds to existing evidence that common genetic influences underly the co-occurrence of DLD and psychopathology. The study also provides preliminary evidence that genetic influences on internalizing problems are stronger in young people with DLD. That is, succeptibility to genetic influences on psychopathology (internalizing problems) may be increased by the presence of DLD. Our findings highlight the need for early identification of young people at risk of DLD who can be specifically targeted to minimize their risk for psychopathology, especially internalizing problems. Future studies should investigate specific mechanisms of these relationships and use larger samples to derive more precise estimates of etiological influences on these relationships.

## Supplemental Material

sj-pdf-1-ldx-10.1177_00222194211019961 – Supplemental material for Developmental Language Disorder and Psychopathology: Disentangling Shared Genetic and Environmental Influences Supplemental material, sj-pdf-1-ldx-10.1177_00222194211019961 for Developmental Language Disorder and Psychopathology: Disentangling Shared Genetic and Environmental Influences by Umar Toseeb, Olakunle Ayokunmi Oginni and Philip S. Dale in Journal of Learning Disabilities
