# Supplementary material for: Developmental Language Disorder and Psychopathology: Disentangling Shared Genetic and Environmental Influences
Source: J Learn Disabil. 2021 Jun 11;55(3):185–99. doi: 10.1177/00222194211019961 (PMC8996291; doi:10.1177/00222194211019961)
Supplement: sj-pdf-1-ldx-10.1177_00222194211019961 – Supplemental material for Developmental Language Disorder and Psychopathology: Disentangling Shared Genetic and Environmental Influences [file sj-pdf-1-ldx-10.1177_00222194211019961.pdf]

JOURNAL OF LEARNING DISABILITIES SUPPLEMENTAL FILE
TABLE S1. Between Group Comparisons of Additive Genetic and Non-Shared Environmental Influences on Internalizing Problems
ARTICLE TITLE: Developmental Language Disorder and Psychopathology: Disentangling Shared Genetic and Environmental Influences

	without DLD				with DLD			
	a^2		e^2		a^2		e^2	
	**1.**	**2.**	**1.**	**2.**	**1.**	**2.**	**1.**	**2.**
1. Internalising Problems (Ch.)	**.63** **[.52, .72]**	—	**.37** **[.28, .48]**	—	**.69** **[.52, .79]**	—	**.31** **[.21, .47]**	—
2. Internalising Problems (Ad.)	**.95** **[.69, 1.19]**	**.69** **[.58, .78]**	.05 [-.19, .31]	**.31** **[.22, .42]**	**1.00** **[.68, 1.40]**	**.79** **[.64, .88]**	-.01 [-.40, .32]	**.21** **[.12, .36]**

Note. DLD = developmental language disorder, Ch. = childhood, Ad. = adolescence. Values represent standardised variance component influences [95% Confidence Intervals]. Bold values indicate that the 95% confidence intervals did not cross zero.
